# Patient journey to a specialist amyotrophic lateral sclerosis multidisciplinary clinic: an exploratory study

**DOI:** 10.1186/s12913-015-1229-x

**Published:** 2015-12-23

**Authors:** M. Galvin, C. Madden, S. Maguire, M. Heverin, A. Vajda, A. Staines, O. Hardiman

**Affiliations:** School of Nursing and Human Sciences, Dublin City University, Dublin 9, Ireland; Academic Unit of Neurology, Trinity Biomedical Sciences Institute, Trinity College Dublin, Dublin 2, Ireland; Department of Neurology, National Neuroscience Centre, Beaumont Hospital, Dublin 9, Ireland

**Keywords:** Patient journey, Amyotrophic lateral sclerosis (ALS), Medical interview, Chart review, Multidisciplinary clinic, Ireland

## Abstract

**Background:**

The multidisciplinary approach in the management of Amyotrophic Lateral Sclerosis (ALS) has been shown to provide superior care to devolved care, with better survival, improved quality of care, and quality of life. Access to expert multidisciplinary management should be a standard for patients with ALS. This analysis explores the patient journey from symptom onset and first engagement with health services, to the initial visit to a specialist ALS Multidisciplinary Clinic (MDC) in Dublin, Ireland.

**Methods:**

A retrospective exploratory multi-method study details the patient journey to the MDC. Data from medical interviews and systematic chart review identifies interactions with the health services and key timelines for thirty five new patients presenting with a diagnosis of ALS during a 6 month period in 2013.

**Results:**

The time from first symptom to diagnosis was a mean of 16 months (median 13 months), with a mean interval of 19 months (median 14.6) from first symptoms to arrival at the MDC. The majority of patients were seen by a general practitioner, and subsequently by neurology services. There was an average of four contacts with health services and 4.8 investigations/tests, prior to their first Clinic visit. On the first visit to the MDC patients are linked into an integrated ‘system’ that can provide specialist care and link with voluntary, palliative and community services as required.

**Conclusions:**

Engagement with a multidisciplinary team has implications for service utilization and quality of life of patients and their families. We have demonstrated that barriers exist that delay referral to specialist services. Comprehensive data recording and collection, using multiple data sources can reconstruct the timelines of the patient journey, which can in turn be used to identify pathways that can expedite early referral to specialist services.

## Background

Amyotrophic Lateral Sclerosis (ALS) is a neurodegenerative disease characterized by progressive degeneration of motor neurons in the brain and spinal cord, and is considered to be one of the most devastating neurological disorders in adults. Up to 50 % of patients with ALS develop a degree of cognitive impairment [1]. There are currently no effective disease modifying therapies for ALS and 70 % of those affected die within three years from symptom onset [2]. There are approximately 110 new cases of ALS in the Republic of Ireland each year, and at least 80 % of these attend the National ALS Clinic in Beaumont Hospital, Dublin [3].

A diagnosis of ALS is primarily based on the physician’s interpretation of clinical symptoms and signs, and investigations to exclude other causes [4–6].The wide range of presentations of ALS coupled with the rapid clinical trajectory require a flexible approach to clinical care that is best provided in an integrated multidisciplinary setting [7, 8]. Early diagnosis and access to expert multidisciplinary management should be available as standard for patients with ALS. However, most studies document a delay of 12-15 months from first symptom to diagnosis, and many patients experience further delays prior to referral to specialist services. The purpose of this study was to document and describe in detail the patient journey from their first symptoms, and first engagement with health services, to their initial visit to the multidisciplinary clinic. The overall objective was to identify possible barriers to accessing multidisciplinary care and highlight the complexity of the referral trajectory.

### Multidisciplinary care

Given the current lack of effective treatment options for ALS, the aim of care is to maximise quality of life from the time of diagnosis through to the end of life. A palliative approach to care, delivered through a multidisciplinary team (MDT) holistically considers the physical, psychological, social and spiritual aspects of the patient, family and illness. Optimal palliative management requires a strategy that integrates hospital-based multidisciplinary care with community-based interventions [9]. The specialist multidisciplinary clinic is more than a bounded spatial and functional entity. It is representative of an approach to clinical management, clinical practice and healthcare delivery. The multidisciplinary approach in the management of ALS has been shown to provide superior care to devolved care, with better survival [7], improved quality of care, and quality of life. Access to expert multidisciplinary management should be a standard for patients with ALS [4, 10].

### ALS Diagnosis

Delays of up to 12 months from the time of first symptoms to diagnosis of ALS have been reported [5]. Andersen et al. [4], report the mean time from the onset of symptoms to confirmation of the diagnosis of ALS of 10–18 months. In England and Wales, Househam and Swash [11], found the mean time from onset of symptoms to diagnosis was 16.2 months, while Donaghy et al*.*, [12] identified a median time 15.6 months from first symptoms to diagnosis in Northern Ireland. A review of diagnosis timelines for patients with ALS over a 20 year period at one treatment centre in the United Kingdom found that the time from the first symptoms experienced by an individual to a definite diagnosis of ALS had remained relatively stable at approximately 12 months [13].

Diagnostic delays are associated with clinical complexity, the patient either not recognizing or denying early or intermittent symptoms, inefficient referral pathways, with patients not being referred to specialist physicians, or being referred to a specialist other than a neurologist/ALS specialist, and the relative rarity and the consequent lack of familiarity with the condition [4, 13, 14]. However, as the disease follows a generally predictable duration from first symptoms to death, such delays represent a significant proportion of the total disease pathway. Early diagnosis has implications for psychological outcomes for patients and their families, for quality of life and service utilization [13, 15]. It reduces the necessity for multiple referrals through the healthcare system in search of a definitive diagnosis, and facilitates future planning and the introduction of Riluzole therapy, currently the only available disease-modifying therapy [4, 16]. The diagnosis story has been described as a sequence of: recognising a problem, seeking medical help, referrals to a series of health professionals, and confirmation of ALS diagnosis [14].

## Methods

During a clinical consultation, the medical history is taken through a semi-structured interview, which has a number of components including presenting complaint, past medical history, current medications, family history, social history, and systems review. Physical examination is also structured by system.This narrative is transcribed into a letter, and sent back to the referring person, and copied to the general practitioner (if they are not the referring doctor), and the patient’s chart.

As part of this study, medical interviews and medical charts for 35 patients who were initial attenders at the MDC were reviewed. These patients had either been previously diagnosed elsewhere or had a confirmation of their diagnosis of ALS on the occasion of this first consultation with the specialist neurologist at the National ALS Centre. Ethical approval was received from Beaumont Hospital Medical Research Ethics Committee review board.

Components of the patient journey derived from a content analysis of the medical interviews, were incorporated into a template. Further data were collected by systematic review of medical charts and by elaboration of the journey using ancillary documentation including nursing notes and those generated by clinical professionals.

**Fig. 1:**
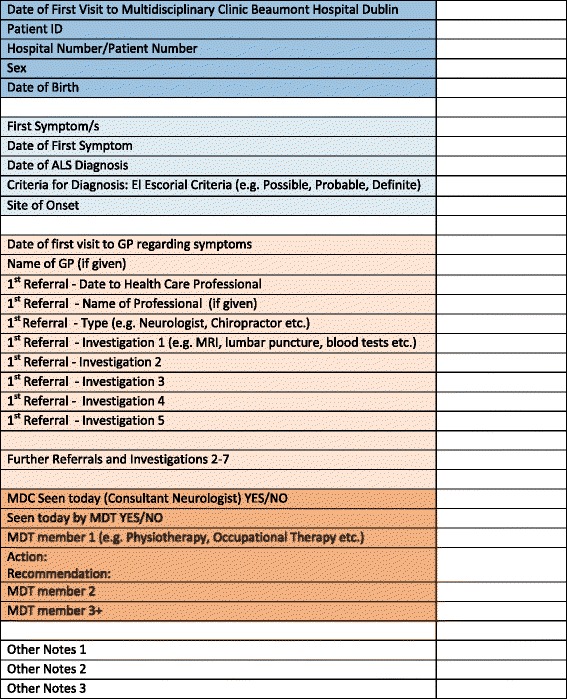
Data Collection Template: chart review – Timeline to Multidisciplinary Clinic

A template (Fig. 1) was designed to collect basic patient information such as administrative identifiers, sex and date of birth. The patient journey itself was documented under a number of headings: first symptoms, site of onset, date of first visit to general practitioner (GP) regarding symptoms, referrals to health care professionals (HCP), interventions or tests performed at each referral destination, date of diagnosis, the initial visit to MDC and related actions and recommendations. The medical charts for this cohort were reviewed for data to populate the timeline template. This was a sequential and iterative process as the data collected in one phase contributed to the data collected in another i.e. components of the patient journey derived from a content analysis of medical interviews, informed the design of the data collection template which subsequently documented the patient journey with data from the medical chart review. The site for this study is a single clinic, at the National ALS Centre at Beaumont Hospital Dublin. A consecutive sample of thirty five new patients presenting with a diagnosis of ALS during a 6 month period in 2013 were included in this analysis.

## Results

From the medical interviews we identified components of the patient’s journey to the MDC. The patient and caregiver/family present described when s/he first started feeling unwell, often noticing symptoms that appeared unrelated while doing ordinary tasks; and sometimes no action was taken as there was no immediate concern. The family member often noted symptoms independently of the patient, encouraging him/her to seek medical advice. The first contact with the health services regarding symptoms was most often with a general practitioner, and then a referral journey began from one health care professional to another, from generalist to specialist, involving a range of tests and interventions.

Findings from the chart review indicate that on first attendance at the MDC the mean age for this group of 35 patients was 64 years (range 38.7 to 75.9), 51 % were male, and 63 % had spinal onset. 51 % of patients were diagnosed as ‘definite’ for ALS according to the El Escorial criteria (Table 1).

**Table 1:** Patient details

Age	Mean (years)		64.3
	Range	38.7 - 75.9	
		*N*	*(%)*
Sex	Male	18	(51.4 %)
	Female	17	(48.6 %)
Site of Onset	Bulbar	8	(22.9 %)
	Bulbar/cognitive	2	(5.7 %)
	Spinal	22	(62.9 %)
	Spinal/cognitive	3	(8.6 %)
El Escorial	Definite	18	(51.4 %)
	Possible	5	(14.3 %)
	Probable	10	(28.6 %)
	Not Stated	2	(5.7 %)

### Patient journey

#### Timeline

**Fig. 2:**
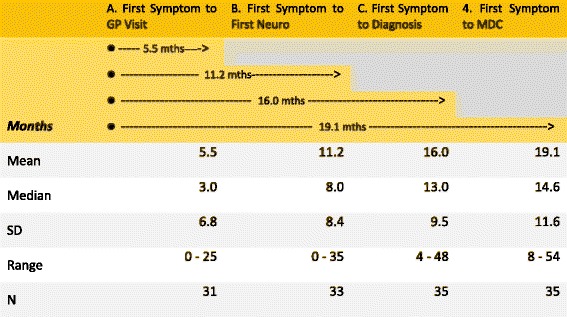
Timeline –First Symptom Timeline

The patient journey from first symptoms, contact with health services, ALS diagnosis and the initial visit to the MDC at Beaumont Hospital is illustrated in Fig. 2 below. There was an average of 5.5 months (median 3 months) between the first symptom and visiting a GP; a mean of 11.2 months (median 8) from the first symptom to contact with Neurology services, and 16 months (median 13) to ALS diagnosis, with over one and a half years (mean 19.1 months, median 14.6) from the first symptom to first visit to the multidisciplinary clinic.

#### Health service contact

**Fig. 3:**
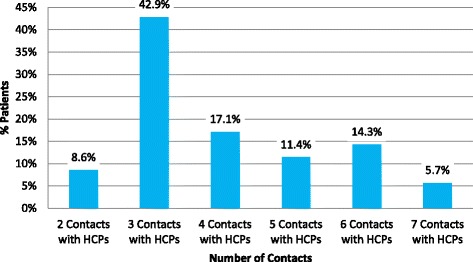
Contacts with Healthcare Professionals

From the information recorded, 91 % of patients (*n* = 32) had contact with a general practitioner before embarking on the subsequent referral pathway to the MDC. For this cohort of patients the time from first GP visit to attending the MDC took an average of 13 months, (median 10.5). All patients had at least two contacts with health care professionals before attending the MDC, with the vast majority having multiple contacts (mean 3.97; median 3) (Fig. 3).

For this group of patients overall there were 139 recorded contacts with health care professionals. The majority (94 %) had contact with some Neurology service (e.g. neurology, neurophysiology, neurosurgery) before attending the MDC, 15 attended Neurology services as a second contact with the health service , 17 as a third contact and so on. 34 % had been to hospital or Accident and Emergency (A&E), and 17 % attended orthopaedic and physiotherapy services respectively (Table 2).

**Table 2:** Sequence of contacts with health services

	Contact sequence						
Professional	1st	2nd	3rd	4th	5th	6th	Total contacts
GP	32				1		33
A&E		2	1				3
Hospital	1	3	4	2		2	12
Neuro		15	17	11	8	5	56
Rheumatology		2					2
ENT*		2		1			3
Other Surgical		1	1		1		3
Physiotherapy	1	4		1			6
Orthopaedic		5	2	1			8
Geriatrician		2	1		1		4
Chiropractor		1	1				2
Other**	1	1	4	1			7

*ENT = Ear Nose and Throat

**e.g. psychiatrist, pain specialist, radiologist

Over the course of these visits to health care professionals there was an average of 4.8 investigations and tests carried out. Neurophysiology and Radiology investigations were the most common (Table 3).

**Table 3:** Investigations Pre MDC

Investigation/Treatment	Number
Neurophysiology (EMG & NCS)	45
Radiology/Imaging (MRI/CT/X-ray/Video fluoroscopy/Neuroimaging)	32
Admission/Contact to hospital	16
Bloods (incl. genetic test and thyroid level)	13
Treatment	10
Got opinion or referred to another speciality	8
Neurological evaluation/cognitive assessment	7
Lumbar puncture	6
Clinical exam	6
Specific tests/investigations	2
Surgery	1
Other	22
Total	168
Total patients	35
Average investigations/treatments pre MDC	4.80

*EMG = Electromyography, NCS = Nerve Conduction Studies, MRI = Magnetic Resonance Imaging, CT = Computed Tomography

The patient journey from first contact with the health services up to initial visit to the multidisciplinary clinic, including numbers of contacts with each service, is illustrated in diagrammatic form in (Appendix 1). A schematic representation of the sequence of health service contacts up to the initial visit to the MDC, and the time taken (in months) from first contact with the health services and the multidisciplinary clinic is shown in (Appendix 2).

### First visit to MDC

**Fig. 4:**
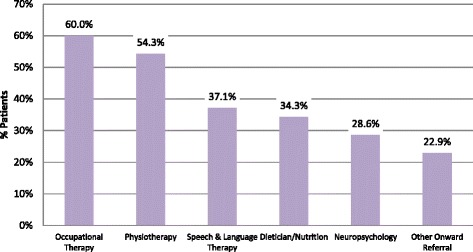
MDT – Service Usage

During the first visit to MDC, these patients were seen by the Consultant Neurologist and Clinical Nurse Specialist and subsequently by other members of the multi-disciplinary team as appropriate to their condition. Occupational Therapy and Physiotherapy were the services used most frequently during this initial visit (Fig. 4). At the Clinic patients received treatment and advice from allied health professionals, with links made with health and social care services in the community and voluntary sector as required.

## Discussion

The personal relationship between the patient and physician remains the basis of high quality treatment [17]. Illness, and the process of being ill, is formed and articulated in the physician-patient encounter. The patient’s experience of symptoms is interpreted by physician’s medical knowledge, leading to a diagnosis, and a corresponding therapeutic intervention [18]. Physicians must know the facts of pathophysiology but also the individual patient and the symptoms, signs and answers to questions that fill out the story of the illness presented to him/her for medical attention. The interpretive reasoning required to understand signs and symptoms and to reach a diagnosis is represented in its situated and circumstantial uncertainty in narrative [19].

The pre-diagnostic phase has been described as a ‘diagnostic roundabout’ [13], characterized by uncertainty and traumatic experiences. From the chart review we identified that the majority of this cohort of patients (91 %) attended a GP as their first contact with the health services. We have identified an average of four contacts with health services (with a maximum of 7 contacts) and 4.8 investigations/tests, prior to the first MDC visit. Neurophysiology and Radiology investigations were the most frequently performed.

The time from first symptom to diagnosis was a mean of 16 months (median 13 months). This may reflect delayed referral for specialist investigations, and patients being initially directed to specialities other than neurology such as physiotherapy, orthopaedics, or to staff in local general hospitals which may not have recognised the underlying neurological problems. Overall, this led to a mean delay of 11.2 months (median 8) from first symptom to contact with Neurology services. The time from first GP visit to attending the MDC took an average of 13 months, (median 10.5 months). The timeline constructed shows a mean interval of 19 months (median 14.6) from first symptoms to arrival at the specialist MDC. The Patient Journey (Appendix 1 and 2) identifies the sequence of health service contacts, and the journey over many months, which patients travelled before the MDC. Patients and their families began their engagement with the multidisciplinary team at their first clinic visit, with a majority being seen by Occupational Therapy and Physiotherapy, and others making contact with Speech and Language Therapy, Dietician and Neuropsychology.

Few studies have employed qualitative approaches to explore the diagnostic experience from the perspective of patients and their carers [14]. As part of a longitudinal study at the National ALS Centre in Dublin, following the patient and caregiver journey, informal caregivers of people with ALS were asked about the time up to the ALS diagnosis and what it was like for the caregiver. A preliminary thematic analysis [20] of their responses provides an insight into the journey to the MDC, as a series of seemingly unrelated symptoms went unrecognised, with subsequent differential diagnosis by health professionals*.* The caregivers recounted stages on the referral journey, as one commented *“we first went to the physiotherapist, then the neurologist about her migraines”,* and for another *“the doctors … as a last resort they sent us to a speech and language therapist and the speech and language therapist referred us to a consultant neurologist.”* For some it seems that health-related information was revealed through less than optimal communication with the HCPs, and according to one caregiver *“the way the doctor told us was not great. Doctor left us and thought we would ask lots of questions and we didn’t ask anything”.*

The medical interview is a version of events as remembered and recounted by the patient and his/her family, mediated by the health care practitioner. This account is reflected in the medical interview/letter and presented in a particular format. It is important to bear in mind the positionality of the patient-caregiver and physician in a power-knowledge-practice complex; and also to consider the social rules of engagement and interaction, the social location of the patient and family member, time constraints, and that the illness narrative can be both formed and changed by the medical encounter (co-creation). Nonetheless, the medical interview provides valuable insight into the crucial journey of the patient and caregiver to their initial visit to the MDC, highlighting contacts with the health services and timelines on the journey. A review of the medical charts for these patients generated fine-grained information from which detailed schematic and individual patient journeys were constructed.

The use of multiple data sources in this analysis - medical interviews and systematic chart review - allowed for cross-checking of information in an iterative process for clarification of details as required. The interviews recorded information in narrative form and exact dates were not always included, while the medical charts contained comprehensive information which was often ‘fleshed out’ by going back to the medical interview data for corroboration and/or context*.*

Through the process of this study gaps in data recording were encountered. During the initial medical interview it is understandable that patient and caregiver/family might not clearly recall detailed information and dates, names or clinical specialties of practitioners whom they encountered, or precise tests and investigations. The precise noting of dates of referrals and tests, in a busy clinical situation is not always possible. For accurate analysis of the patient journey it is important to record detailed and specific information and to generate robust data for health services research. This study has contributed to highlighting the implications of data recording for clinical management leading to the development of a systematic recording and data collection framework.

Access to multidisciplinary care should be the standard for all ALS patients. Our findings suggest a mean of 19 months from first symptom to the initial visit to the Clinic, during which time the patient attends a variety of health care practitioners and undergoes tests and investigations as directed. The timelines on the journey from noticing first symptoms and involvement with health services to arrival at the MDC, can involve personal and systemic factors. Patients may ignore or choose to deny symptoms, and postpone engagement with health care professionals, while obstacles within the health system such as shortages in essential staff, poor access to investigations, limited knowledge of the condition and a failure to recognize the benefits of the MDC can affect the trajectory of the subsequent journey to the MDC. While earlier diagnosis could avoid unnecessary referrals and facilitate future planning, this is not always feasible. ALS is relatively rare and most general practitioners have limited experience of the condition. Our data illustrate that interaction with the general practitioner is a common first point of engagement with the health services. A “red flag” guideline has been established for general practitioners in the UK, the aim of which is to expedite referral for differential diagnosis [21]. Timely referral to the specialist MDC for those with suspected disease is also essential. Our data would suggest that utilization of unnecessary investigations, and an apparent reluctance by non-specialists to make a definitive diagnosis may also delay referral to MDC. Further work using larger patient cohorts will be required to confirm these observations.

This is a small scale study referencing a single specialist clinic. Nevertheless, this exploratory study has identified the importance of comprehensive data collection and the utility of multiple data sources, for the elucidation of touch-points, timelines and identification of the patient journey. Additional information, for example, on patient’s distance from the MDC, socio-economic characteristics and progress of the condition and a larger sample size will allow for the exploration of trends and associations within the data.

## Conclusion

This exploratory multi-method study details the patient journey to a specialist ALS Multidisciplinary Clinic (MDC). On the first visit to the MDC, patients are linked into an integrated system comprising expertise in neurology, specialist nursing, respiratory medicine, physiotherapy, occupational therapy neuropsychology and palliative care that can provide specialist care and link with voluntary, palliative and community services as required.

In ALS the precise patient journey from first symptom to diagnosis to death and the economic costs of disease management have not yet been fully mapped. The notion of a pathway conveys the idea of a linear progression albeit one that can split off in a number of directions. The concept of a journey is preferred here and captures an impression as outlined in the patient narratives as something that is embarked upon without a known destination in mind. This analysis has tracked the patient journey to the Multidisciplinary Clinic at the National ALS Centre in Dublin. Engagement with a multidisciplinary team has implications for service utilization and quality of life of patients and their families. Detailed analysis of the patient journey is urgently required by following the journeys of people in real time from the point of diagnosis, and by retrospective collection of experiences prior to diagnosis. This method can generate a comprehensive map of the interplay between the patient/caregiver dyad with the health services, and in doing so can formulate a coherent set of guidelines that will facilitate early access to specialist services.

## Appendix 1

Patient Journey from first contact with health services to MDC – (numbers indicate contacts with each service)

## Appendix 2

Schematic representation of Patient Journey to MDC (duration in months from recorded first health service contact to MDC)

## Abbreviations

A&E: accident and emergency
ALS: amyotrophic lateral sclerosis
CT: computed tomography
EMG: electromyography
ENT: ear nose and throat
GP: general practitioner
HCP: Health Care Professionals
MDC: multidisciplinary clinic
MDT: Multidisciplinary Team
MRI: magnetic resonance imaging
NCS: nerve conduction studies

## References

[1] Elamin M, Bede P, Byrne S, Jordan N, Gallagher L, Wynne B (2013). Cognitive changes predict functional decline in ALS a population-based longitudinal study. Neurology.

[2] Rooney J, Byrne S, Heverin M, Corr B, Elamin M, Staines A et al. Survival Analysis of Irish Amyotrophic Lateral Sclerosis Patients Diagnosed from 1995-2010. Plos One. 2013;8(9). doi:10.1371/journal.pone.0074733. 10.1371/journal.pone.0074733 PMC3786977 24098664

[3] O’Toole O, Traynor BJ, Brennan P, Sheehan C, Frost E, Corr B (2008). Epidemiology and clinical features of amyotrophic lateral sclerosis in Ireland between 1995 and 2004. J Neurol Neurosurg Psychiatry.

[4] Andersen PM, Abrahams S, Borasio GD, de Carvalho M, Chio A, Van Damme P (2012). EFNS guidelines on the Clinical Management of Amyotrophic Lateral Sclerosis (MALS) - revised report of an EFNS task force. Eur J Neurol.

[5] Hardiman O, van den Berg LH, Kiernan MC (2011). Clinical diagnosis and management of amyotrophic lateral sclerosis. Nat Rev Neurol.

[6] McDermott CJ, Shaw PJ (2008). Diagnosis and management of motor neurone disease. BMJ.

[7] Rooney J, Byrne S, Heverin M, Tobin K, Dick A, Donaghy C (2015). A multidisciplinary clinic approach improves survival in ALS: a comparative study of ALS in Ireland and Northern Ireland. J Neurol Neurosurg Psychiatry.

[8] Traynor B, Alexander M, Corr B, Frost E, Hardiman O (2003). Effect of a multidisciplinary amyotrophic lateral sclerosis (ALS) clinic on ALS survival: a population based study, 1996–2000. J Neurol Neurosurg Psychiatry.

[9] Bede P, Oliver D, Stodart J, van den Berg L, Simmons Z, Brannagain DO (2011). Palliative care in amyotrophic lateral sclerosis: a review of current international guidelines and initiatives. J Neurol Neurosurg Psychiatry.

[10] Van den Berg JP, Kalmijn S, Lindeman E, Veldink JH, de Visser M, Van der Graaff MM (2005). Multidisciplinary ALS care improves quality of life in patients with ALS. Neurology.

[11] Househam E, Swash M (2000). Diagnostic delay in amyotrophic lateral sclerosis: what scope for improvement?. J Neurol Sci.

[12] Donaghy C, Dick A, Hardiman O, Patterson V (2008). Timeliness of diagnosis in Motor Neurone Disease: a population-based study. Ulster Med J.

[13] Mitchell JD, Callagher P, Gardham J, Mitchell C, Dixon M, Addison-Jones R (2010). Timelines in the diagnostic evaluation of people with suspected amyotrophic lateral sclerosis (ALS)/motor neuron disease (MND)–a 20-year review: Can we do better?. Amyotroph Lateral Scler.

[14] O’Brien MR, Whitehead B, Jack BA, Mitchell JD (2011). From symptom onset to a diagnosis of amyotrophic lateral sclerosis/motor neuron disease (ALS/MND): experiences of people with ALS/MND and family carers - a qualitative study. Amyotroph Lateral Scler.

[15] Bromberg M (1999). Accelerating the diagnosis of amyotrophic lateral sclerosis. Neurologist.

[16] Shaw PJ, Wood-Allum C (2010). Motor neurone disease: a practical update on diagnosis and management. Clin Med.

[17] Larson EB, Yao X (2005). Clinical empathy as emotional labor in the patient-physician relationship. Jama-Journal of the American Medical Association.

[18] Kalitzkus V, Matthiessen PF (2009). Narrative-based medicine: potential, pitfalls, and practice. Perm J.

[19] Montgomery K. How doctors think: Clinical judgment and the practice of medicine. Oxford University Press; New York 2006.

[20] Braun V, Clarke V (2006). Using thematic analysis in psychology. Qual Res Psychol.

[21] Motor Neurone Disease Association and Royal College of General Practitioners. Red flag diagnosis tool 2014. http://www.guidelines.co.uk/central_nervous_system_mnda_mda#.Vh6l9Csjnzg. Accessed 14^th^ October 2015

